# Supplementary material for: Human genetic variants and age are the strongest predictors of humoral immune responses to common pathogens and vaccines
Source: Genome Med. 2018 Jul 27;10:59. doi: 10.1186/s13073-018-0568-8 (PMC6063007; doi:10.1186/s13073-018-0568-8)
Supplement: Supplementary file 2 — Figure S1. Principal Component Analysis. Figure S2. Distribution of serological variables, and clinical thresholds. Figure S3. Seroprevalence data. Figure S4. Impact of non-genetic factors on serostatus. Figure S5. Evolution of serostatus with age and sex. Figure S6. Correlations between age and IgG specific to Rubella and T. gondii. Figure S7. QQ plots for logistic regressions preformed in the study. Figure S8. QQ plots for linear regressions preformed on total Ig levels. Figure S9. QQ plots for linear regressions preformed for pathogen-specific IgG levels. Figure S10. QQ plots for burden testing analyses preformed for all binary phenotypes. Figure S11. QQ plots for burden testing analyses preformed for total Ig levels. Figure S12. QQ plots for burden testing analyses preformed for pathogen-specific IgG levels. (DOCX 89996 kb) [file 13073_2018_568_MOESM2_ESM.docx]

**Fig.S1 Principal Component Analysis of the *Milieu Intérieur* cohort**

**
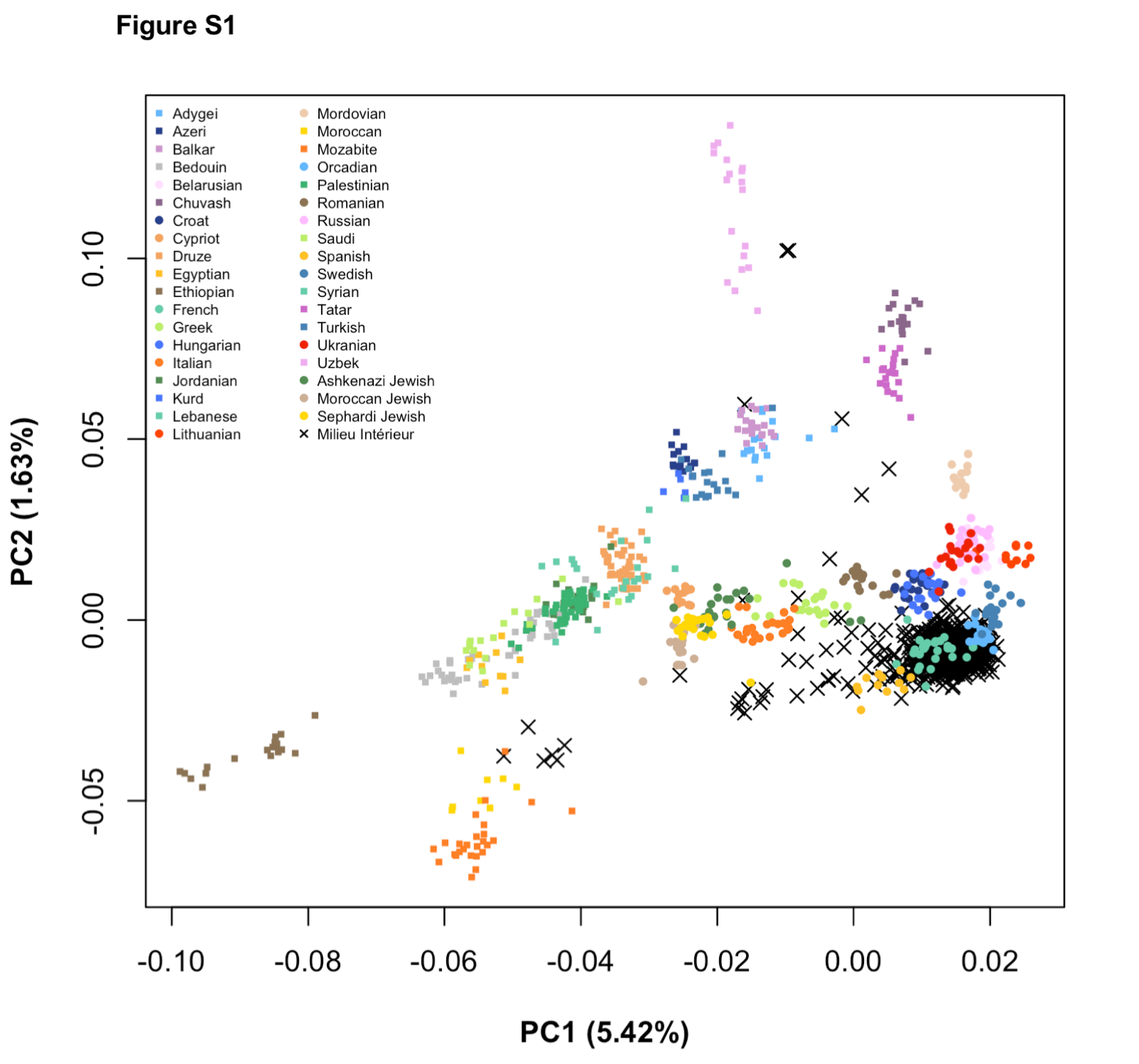
**

Adapted from [16], the genetic structure of the study population was estimated using principal component analysis (PCA).

**Fig.S2 Distribution of serological variables, and clinical threshold used for determination of serostatus.**


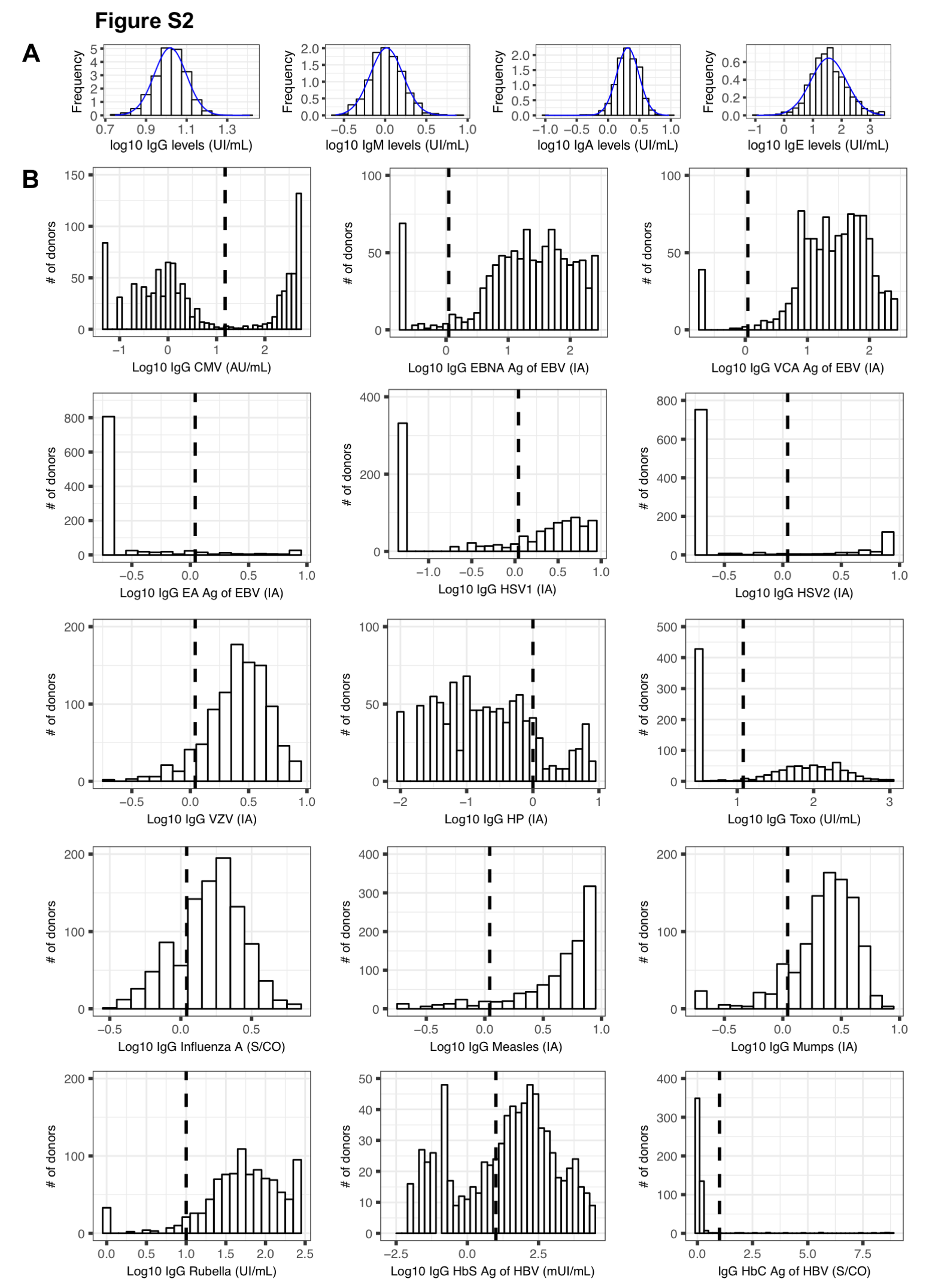


(**A**) Distribution and probability density curve of Log10-transformed IgG, IgM, IgA, IgE levels in the 1,000 study participants. (**B**) Distribution of Log10-transformed antigen-specific IgG levels. The vertical lines indicate the clinical threshold determined by manufacturer, and used for determining the serostatus of the donors for each serology.

**Fig.S3 Seroprevalence data in the 1,000 healthy donors.**


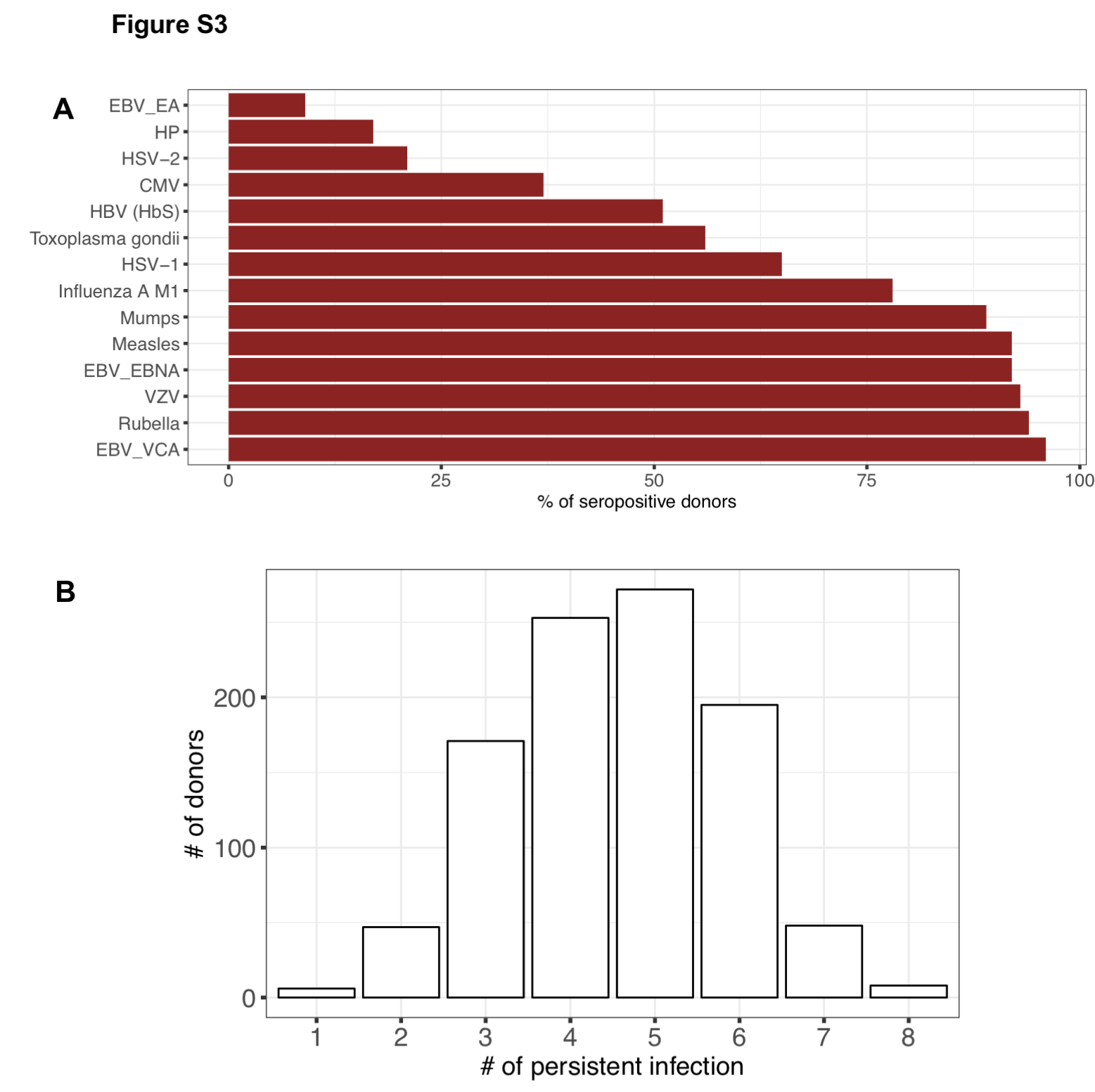


(**A**) Percentage of seropositive donors for each indicated serology in the *MI* study (for HBV serology, percentages of anti-HBs IgGs are indicated). (**B**) Distribution of the number of positive serologies in the 1,000 healthy donors regarding the 8 persistent or recurrent infections tested in our study (*i.e.* CMV, Influenza, HSV1, HSV2, TP, EBV_EBNA, VZV, HP).

**Fig.S4 Impact of non-genetic factors on serostatus.**


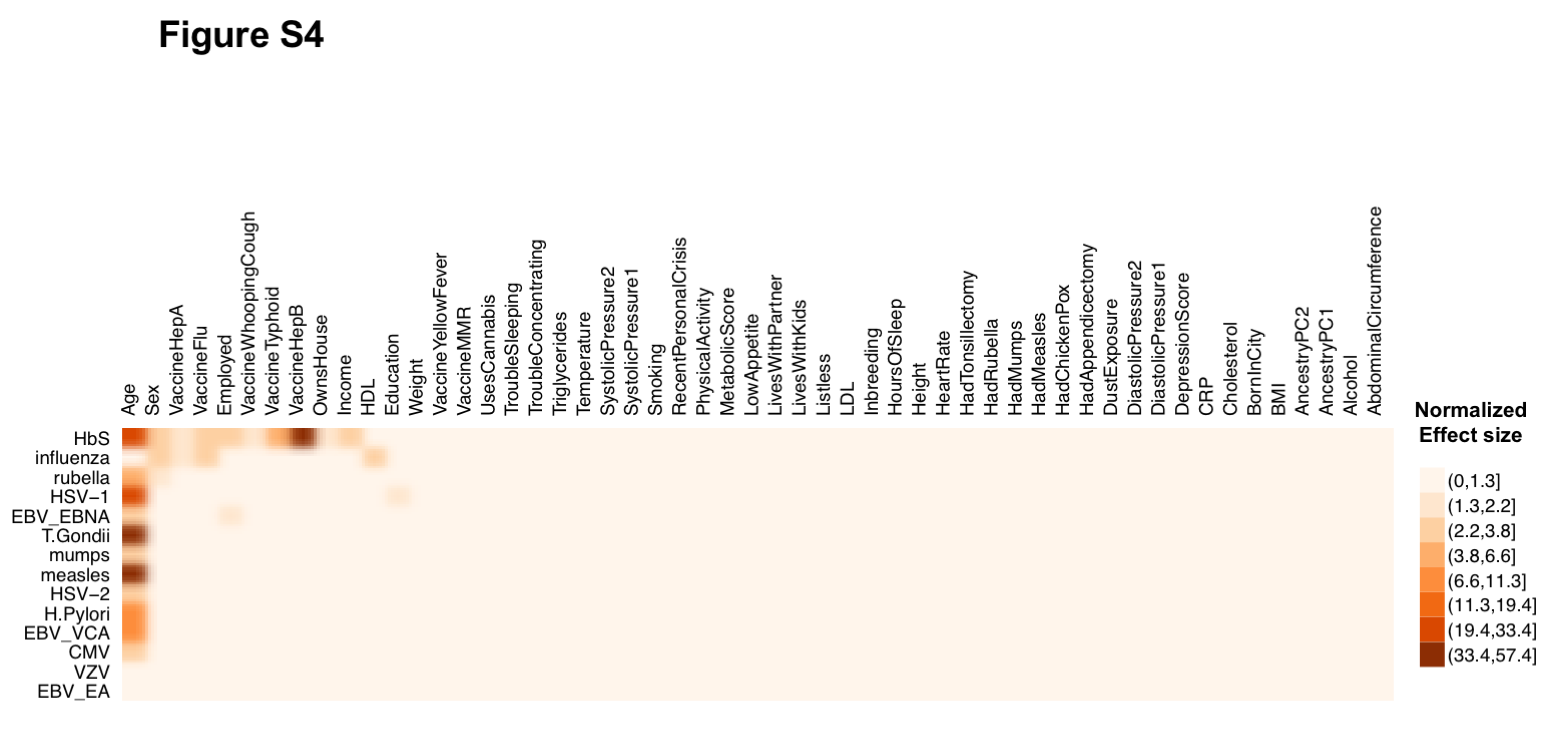


Adjusted *P*-values (FDR) of the large-sample chi-square likelihood ratio tests of effect of non-genetic variables on serostatus, obtained from mixed models.

**Fig.S5 Evolution of serostatus with age and sex.**

(**A**) Effect sizes of significant quadratic age terms (adjusted *P*-values (adj. *P*<0.05)) on serostatus. Effect sizes were estimated in a generalized linear mixed model, with serostatus as response variable, and age and sex as treatment variables. This model includes both scaled linear and quadratic terms for the age variable (model = glm(y~Age+I(Age^2)+Sex, family=binomial)). Here results of quadratic age term are represented. Scaling was achieved by centering age variable at the mean age. All results from this analysis are provided in Additional File 1: Table S5. Dots represent the mean of the beta. Lines represent the 95% confidence intervals. (**B-P**) Odds of being seropositive for each of the 15 antigens considered in our study, as a function of age in men (blue) and women (red). Indicated *P*-values were obtained using a logistic regression with Wald test, with serostatus binary variables (seropositive versus seronegative) as the response, and age and sex as treatments. (**Q**) Effect sizes of significant sex-age interaction term (adjusted *P*-values (adj. *P*<0.05)) on serostatus. Effect sizes were estimated in a generalized linear mixed model, with serostatus as response variable, and age and sex as treatment variables. This model includes age and sex variable, as well as age*sex interaction term (model = glm(y~Age+Sex+Age*Sex, family=binomial)). Here results of the interaction term are represented. Scaling was achieved by centering age variable at the mean age. All results from this analysis are provided in Additional File 1: Table S5. Dots represent the mean of the beta. Lines represent the 95% confidence intervals.

**Fig.S6 Correlations between age and IgG specific to Rubella and T. Gondii.**


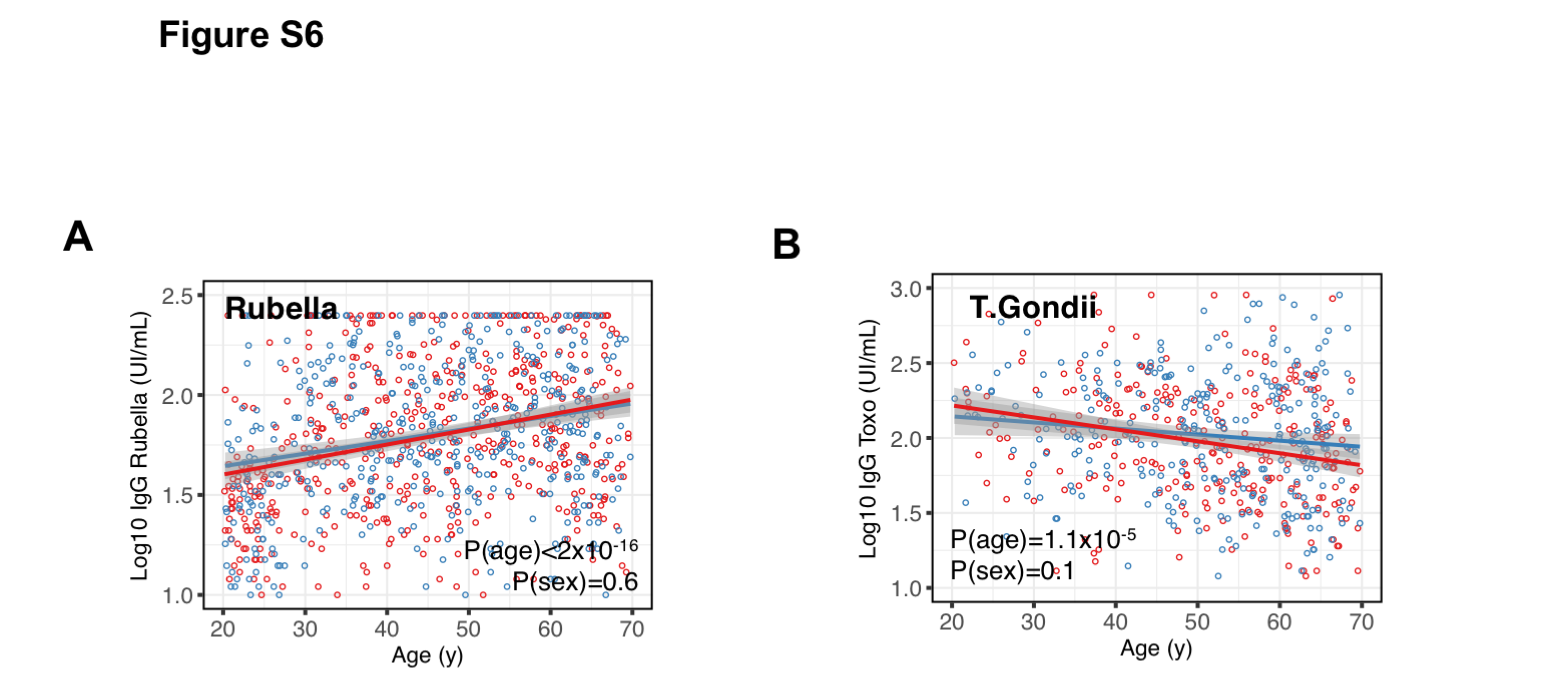


(**A**) Relationships between Log10-transformed anti-rubella IgGs, and (**B**) Log10-transformed anti-Toxoplasma gondii IgGs (right) and age. Regression lines were fitted using linear regression. Indicated adj. *P* were obtained using the mixed model and corrected for multiple testing using the FDR method.

**Fig.S7 Assessment of false positive inflation rates for all genome-wide logistic regressions preformed in the study.**


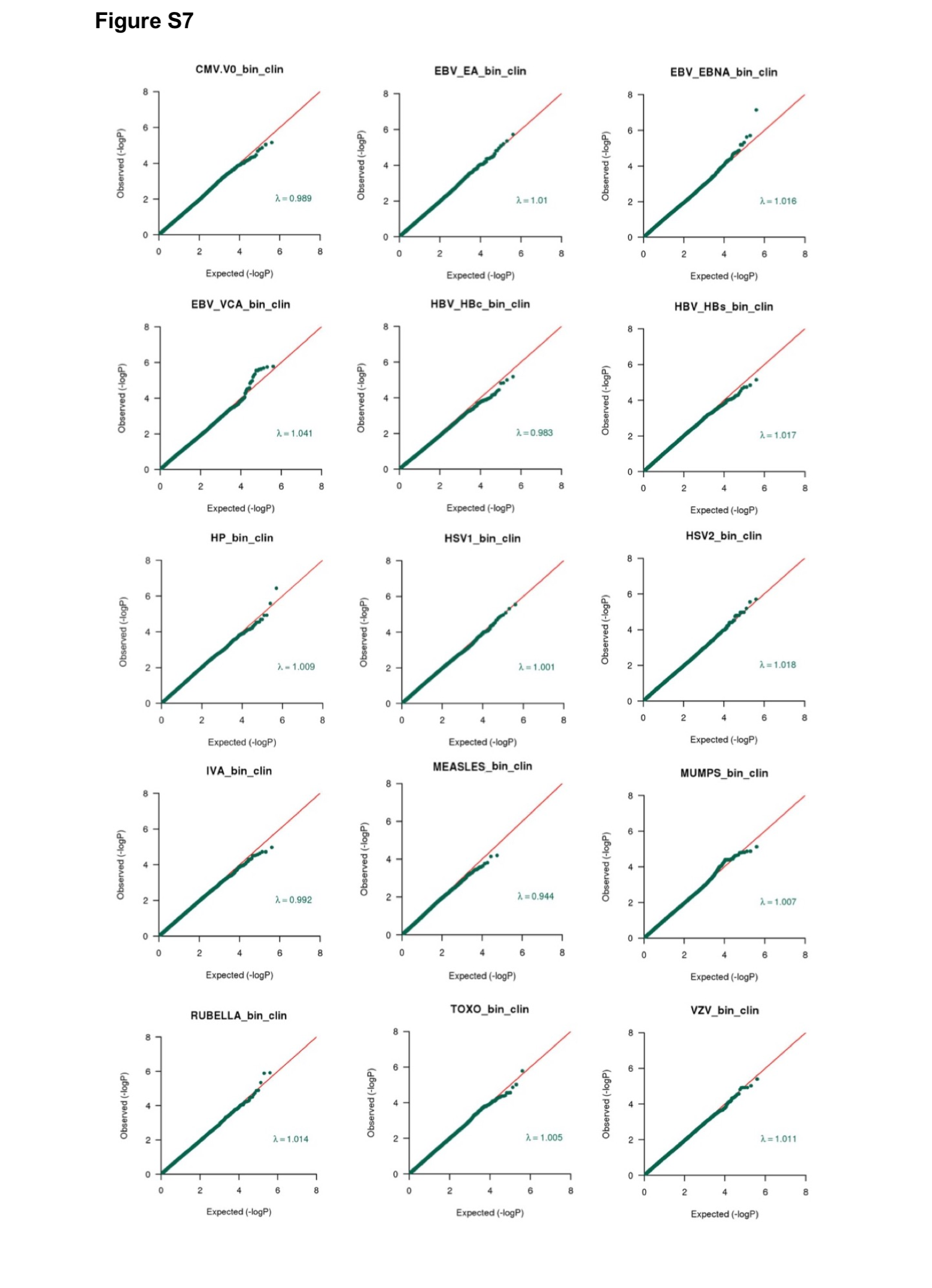


The quantile-quantile plots and lambda values of all genome-wide logistic regressions preformed in the study.

**Fig.S8 Assessment of false positive inflation rates of genome-wide linear regressions preformed for total immunoglobulin levels.**


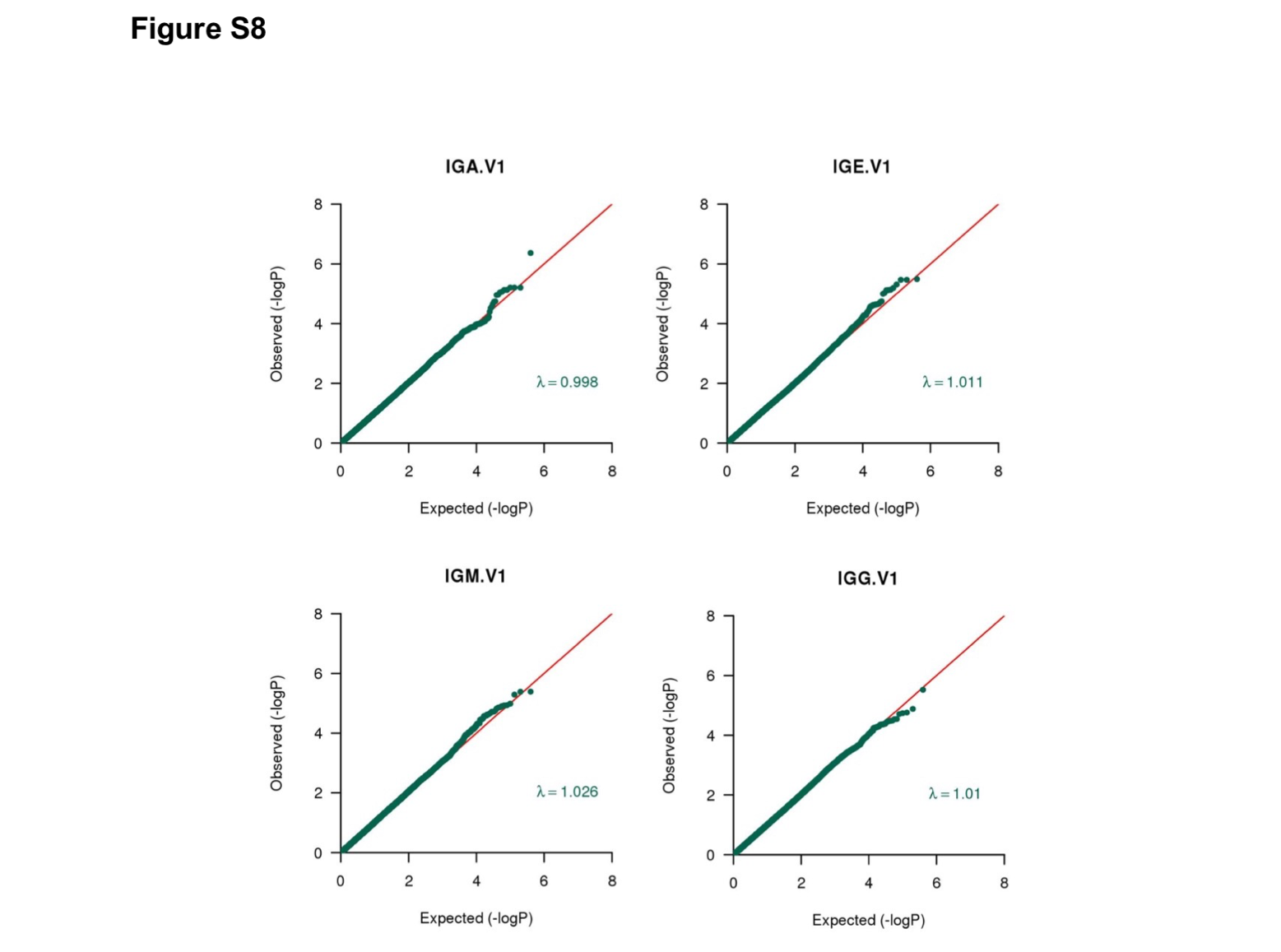


The quantile-quantile plots and lambda values of genome-wide linear regressions preformed for total immunoglobulin levels.

**Fig.S9 Assessment of false positive inflation rates of genome-wide linear regressions preformed for pathogen-specific IgG levels.**


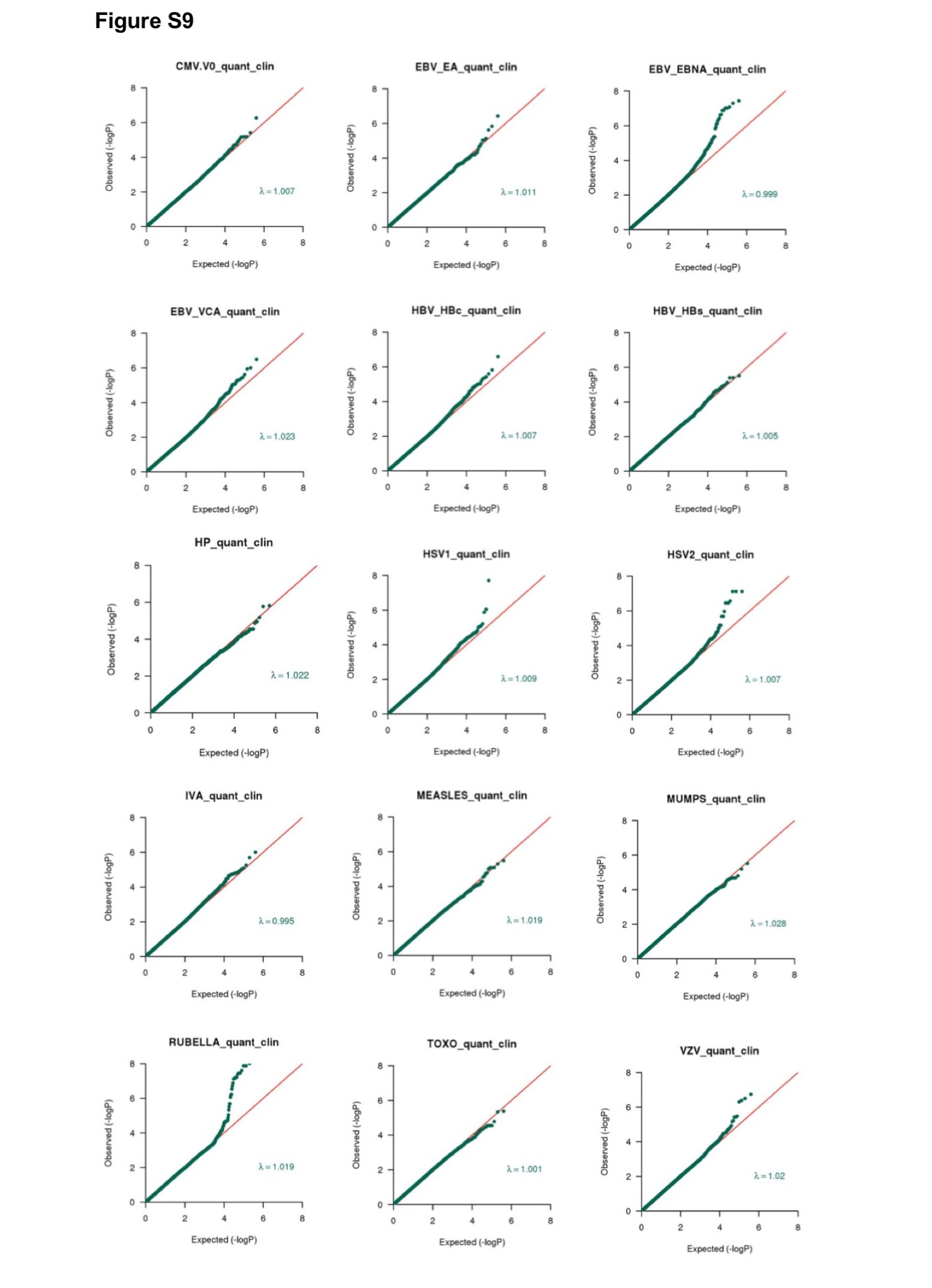


The quantile-quantile plots and lambda values of genome-wide linear regressions preformed for pathogen-specific IgG levels.

**Fig.S10 Assessment of false positive inflation rates for burden testing analyses preformed for all binary phenotypes in the study.**

**
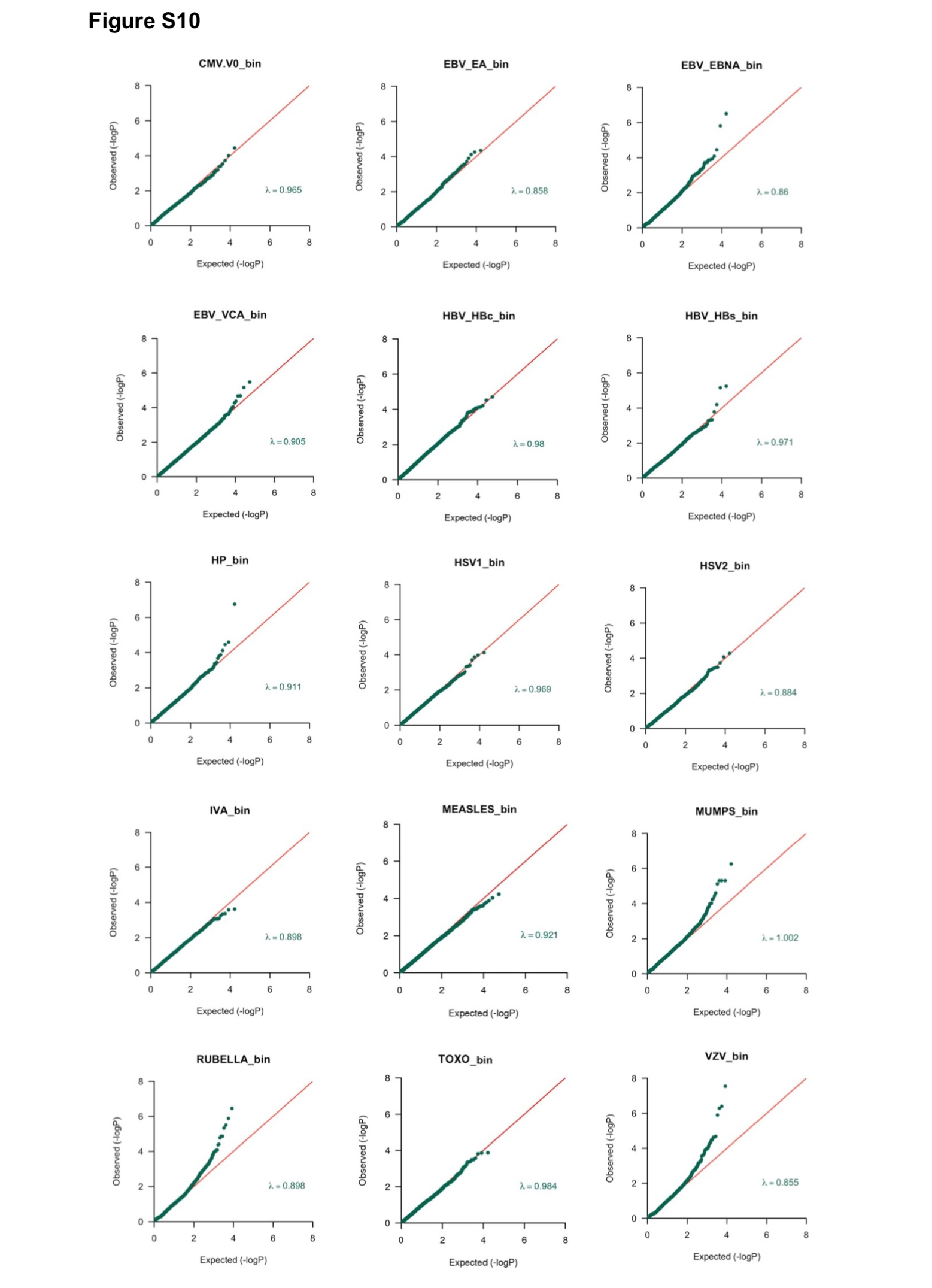
**

The quantile-quantile plots and lambda values for burden testing analyses preformed for all binary phenotypes in the study.

**Fig.S11 Assessment of false positive inflation rates for burden testing analyses preformed for total immunoglobulin levels.**

**
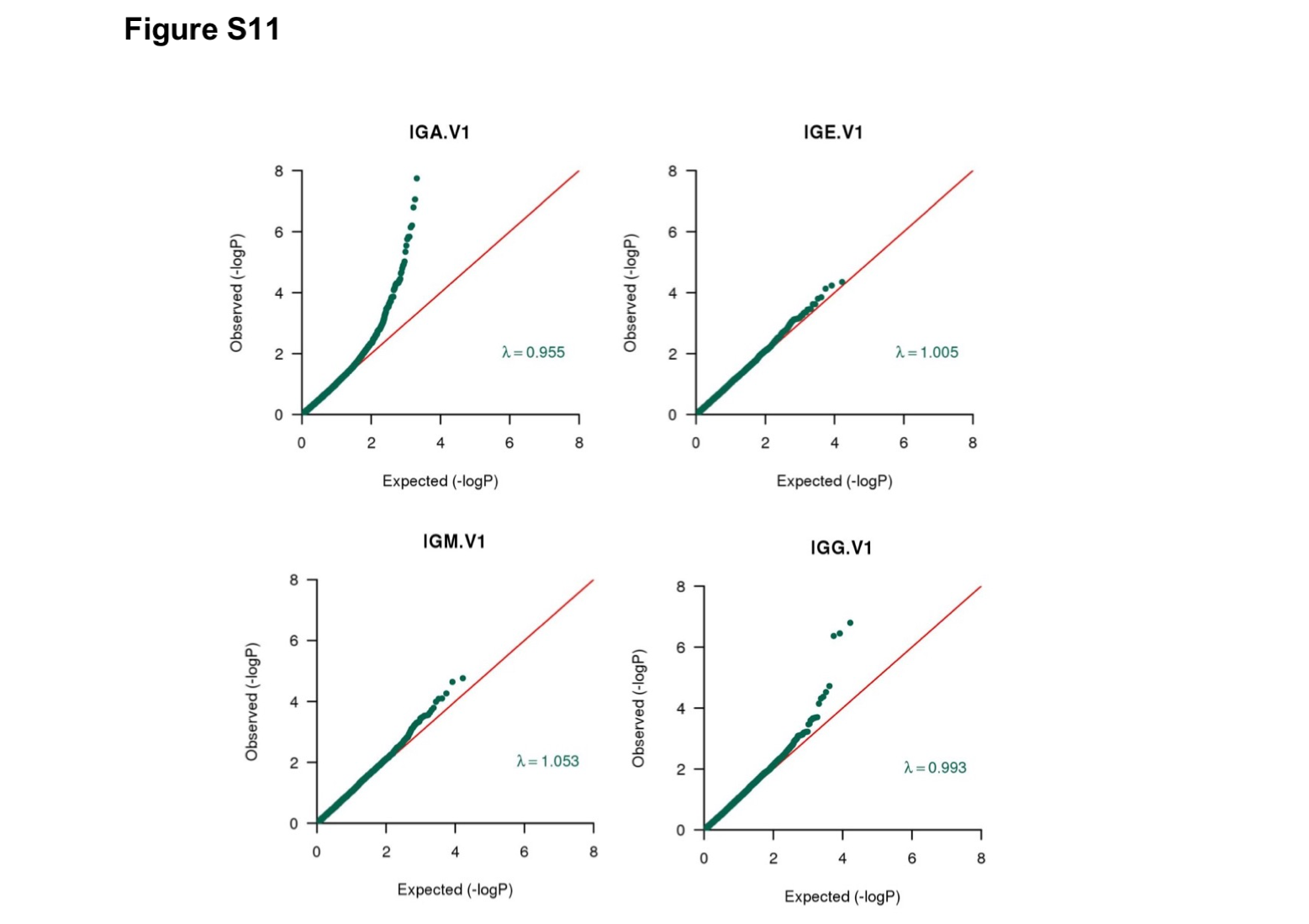
**

The quantile-quantile plots and lambda values for burden testing analyses preformed for total immunoglobulin levels.

**Fig.S12 Assessment of false positive inflation rates for burden testing analyses preformed for pathogen-specific IgG levels.**

**
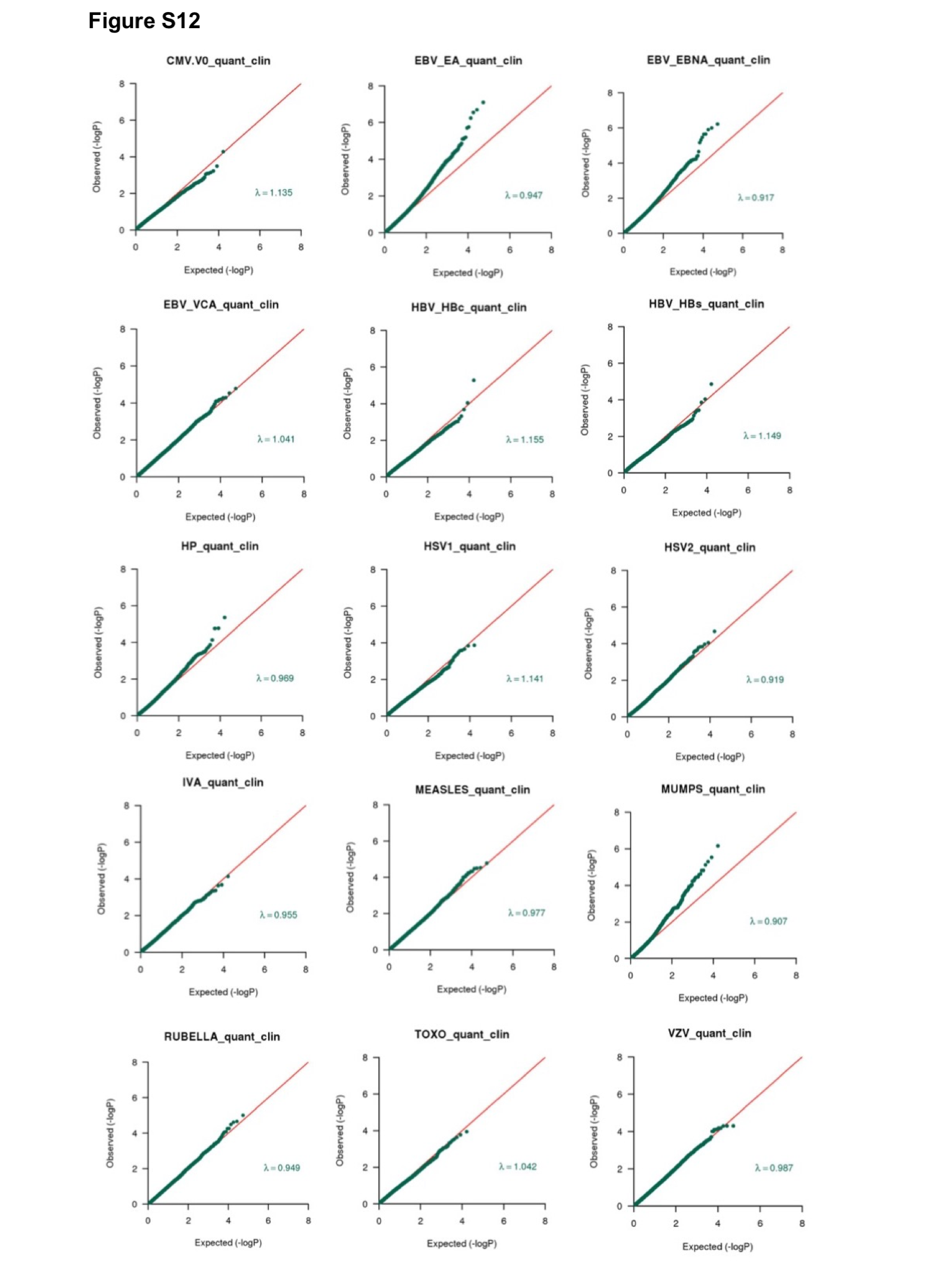
**

The quantile-quantile plots and lambda values for burden testing analyses preformed for pathogen-specific IgG levels.
